# Generalizable gesture classification of HDsEMG using volume representations of muscles averaged across multiple individuals

**DOI:** 10.1038/s41598-025-28215-y

**Published:** 2025-11-21

**Authors:** Jonathan Lundsberg, Anders Björkman, Nebojsa Malesevic, Christian Antfolk

**Affiliations:** 1 Department of Biomedical Engineering, Faculty of Engineering, Lund University, Lund, Sweden; 2 Institute of Clinical Sciences, Sahlgrenska Academy, University of Gothenburg, Gothenburg, Sweden; 3 Department of Hand Surgery, Sahlgrenska University Hospital, Mölndal, Sweden

**Keywords:** High-density sEMG, Localization, Muscle modelling, Gesture classification, Motor control, Biophysical models, Neural decoding, Biomedical engineering

## Abstract

Human hands can perform far more gestures than the number of muscles controlling them, as most gestures result from coordinated combinations of muscle activations and relaxations. This complexity poses a key challenge for human-machine interfaces performing gesture classification based on electromyography (EMG). Rather than identifying all conceivable gestures, it may be simpler to instead identify the activity of the individual muscles which generate a variety of complicated gestures. Here we suggest a three-dimensional model with volume representations of individual digit extensor muscles, averaged across multiple individuals, and evaluate its application and performance in hand gesture classification. Time-domain peaks in high-density surface EMG data from different hand gestures were extracted and localized within the model, from which a gesture classification scheme was generated for both single and multi-label cases. The model was created and tested on a publicly available dataset with 19 participants, leveraging a leave-one-out approach to assess inter-subject generalizability, and multi-label data to assess generalizability to gestures not included in the creation of the model. For single-label classification performance, true positive rates were between 61.9 and 95.1%, with false positive rates between 0 and 24.1%, for different single-digit extensions. The multi-label test demonstrated some degree of generalizability in identifying completely new gesture compositions, while simultaneously maintaining the leave-one-out approach for inter-subject generalizability. A model generated with this approach could be used for gesture classification by anyone, without individual modelling data, with the potential to generalize to any number of gesture compositions.

## Introduction

The human hand can perform a large number of actions despite being controlled by a limited number of intrinsic and extrinsic muscles. While the anatomy of forearm and hand muscles is well understood, the permutations of muscle combinations that generate grasps, gestures, and actions are vast, which poses a challenge for the development of advanced human-machine interfaces (HMIs). HMIs based on electromyography (EMG) decode descending neural activity from neurophysiological signals generated by muscles^1–3^. These signals are processed by various algorithms to interpret user intent, enabling control of computer interfaces or devices such as prosthetic limbs.

There are broadly two categories of control algorithms for HMIs based on either discrete categorisation (classification) of, e.g., hand gestures^4,5^ or continuous estimation (regression) of, e.g., muscle force^6^. Advanced gesture classification algorithms commonly involve learning features unique to each gesture from training data. However, unravelling EMG signals for all possible hand gestures requires a prohibitive amount of training data and a large number of gesture classes, when applying a direct one-to-one approach. Identifying the contractions of all individual muscles, rather than all conceivable gestures, may result in simpler processing of EMG signals, which is an approach worth exploring. Although, it necessitates an understanding of the underlying anatomy and physiology of muscles, and a method which effectively utilizes such knowledge.

A motor unit is a collection of muscle fibres that discharge action potentials in unison along with a motor neuron controlling them. The simultaneous discharge from muscle fibres of the same motor unit generates a compound signal referred to as the motor unit action potential (MUAP). This compound signal amplifies the output of individual motor neurons, providing a direct link to the nervous system and the smallest discrete units of voluntary muscle control. The recorded EMG signal can be seen as a weighted sum of MUAPs, that varies depending on the position of the electrodes and the type of EMG.

EMG can be recorded intramuscularly (iEMG) or from the skin surface (sEMG). While iEMG can provide clear MUAP recordings, and is therefore used clinically to diagnose neurological disorders^7^, its invasiveness limits its use in HMIs. Since needle or wire electrodes record only a few spatially local motor units each^7,8^, a comprehensive muscle-controlled interface would require many needles or wires, leading to significant discomfort for users. Non-invasive sEMG is a much more attractive alternative for generalized or widespread HMIs. However, sEMG suffers from reduced interpretability since the recording is less selective compared to iEMG^7,8^. The recording contains an unknown number of active motor units with superimposed MUAPs, making it more difficult to decode into user intent. The signal interpretability of sEMG is further exacerbated by low-pass filtering effects of the tissue which increases both the overlap and homogeneity of MUAP waveforms^7,9^. On the other hand, the simplicity of sEMG recordings have enabled high-density sEMG (HDsEMG), using large grids of electrodes, to become commonplace in research settings^9,10^. High-density electrode grids generate additional spatial information on MUAPs by recording them from many different positions and angles^11^. Although this additional spatial information is important, a large number of EMG channels also complicate the decoding task; HDsEMG contains too much information, which may or may not be relevant for a given task. Thus, effective processing algorithms are essential to simplify the data and extract meaningful features for robust signal interpretation.

Feature extraction techniques for EMG signals are commonly divided into time-domain, frequency-domain, and time-frequency-domain features^12–14^. Time-domain features are directly computed from signal amplitudes, such as root mean square (RMS), zero crossings, slope sign changes, or waveform length, which makes them computationally efficient and easy to implement. Frequency-domain features, such as mean or median frequency, and time-frequency-domain features, such as a wavelet transform or short-time Fourier transform, are computationally more complex than time-domain features but have been used to study, e.g., muscle fatigue^15,16^. Common classification approaches for extracted features include linear discriminant analysis^17^ and support vector machines^18^, which are robust and simple to use compared to more advanced neural network approaches^19^. Neural networks are promising due to their ability to capture non-linear relationships, which may be present when multiple muscles contract; however, they are severely limited by slow and complicated training, requiring large amounts of training data, limiting the speed of model improvements. While this does not affect a complete model during inference, it can be detrimental when re-training is required, e.g., due shifting contraction patterns over time. Neural networks also lack in explainability, which is important for understanding why estimation errors occur and can limit the analysis of results. While some models show a degree of explainability, they are more difficult to comprehend than models which directly reflect the underlying anatomy. Notably, these established approaches do not explicitly incorporate anatomical or physiological knowledge, such as muscle structure, spatial position or motor unit features.

In contrast to the previously mentioned global EMG features, more recent developments in HDsEMG processing have enabled the study of motor unit characteristics^20,21^, using decomposition algorithms often based on independent component analysis^22–26^. These algorithms make use of known motor unit physiology, i.e. the statistical sparsity of motor unit discharges, to identify their firing patterns, which can then be used to estimate muscle force^20,27–29^, thus directly decoding user intent from the output of the nervous system. However, current decomposition algorithms identify only a fraction of active motor units, whose total number is unknown^27^. Motor unit characteristics therefore lack robustness as features in control algorithms.

We recently proposed a new method which was used to model extensor muscles as ellipsoid volumes from HDsEMG recordings of the forearm during extensions of individual fingers^30^. The spatial information of HDsEMG was utilized to localize individual time-domain peaks in the recording. The three-dimensional distributions of localized peaks were then used to generate the shapes of the ellipsoid volumes. In that study, we found that these muscle representations could be used to determine which muscle volume new peaks belonged to and thus identify muscle activity. However, the study was limited to one dataset with 10 participants performing single digit movements. Furthermore, volume modelling and the assessment of new peaks was performed on the same subject and the same session. As such, the generalizability of this modelling approach remains untested. Generalizability can broadly be broken down into multiple categories, depending on the type of training data required to create a model or, e.g., train a neural network. Cross-session generalizability requires that the algorithm works on data from a new session, with training data from a separate recording; the method needs to adapt to potential variations in electrode placement and data quality. Cross-subject generalizability requires that the algorithm works for completely new subjects not included in the training data; this remains a difficult task with methods typically requiring some amount of individualization or calibration. A final category is generalizability to entirely new gestures not included in the training set, which is rarely explored at all. However, we hypothesize that representative volume modelling of individual muscles in the forearm can generalize to more complex gestures which are a composition of these individual muscles in the model, removing the need for training data from all conceivable gestures. This volume modelling approach is also explainable, in the sense that each volume represents one muscle, which can be contrasted with the known underlying anatomy, and discrepancies in positional estimates can be intuitively understood. Thus, a control scheme based on this model would be highly intuitive, explainable, and generalizable, and therefore worth exploring.

This study investigates the use of representative volume modelling for gesture classification, focusing on its generalizability across subjects and its ability to recognize entirely new gestures not included in the modelling phase. We apply the method described in ^30^ to a publicly available HDsEMG dataset^31^ to create volume representations of individual digit extensor muscles and generate a three-dimensional average human model of the involved extensor muscles in the forearm. A classification scheme is proposed for single and multi-digit gestures based on localization of time-domain extracted EMG peaks within the model. True and false positive classification rates are analysed in single and multi-label cases to assess the method for generalizability across subjects and completely new gesture compositions.

## Method

### Dataset

In this study, the publicly available HDsEMG dataset “Hyser” ^31^ was used. This dataset was chosen for its large coverage of the forearm with monopolar recordings, and the inclusion of single digit gestures. The dataset consists of 256 channels of monopolar HDsEMG, using two adjacent 8-by-8 electrode grids along the anterior side of the forearm as well as the posterior side of the forearm, recording from 20 able-bodied participants performing 34 separate gestures. For single digit gestures, only extensions of the thumb and fingers were recorded in this public dataset, which meant that volume modelling of the individual finger flexors could not be done. Thus, for the purposes of volume modelling of individual muscle regions described in this paper, only the grids on the posterior side were used, primarily covering the extensor digitorum communis (EDC) and extensor pollicis longus (EPL). Additionally, one of the subjects (subject 4) had no recording for one of the single digit gestures and was therefore also excluded. Furthermore, wrist gestures were excluded in order to simplify the model and enable a comparison to our previous study^30^. This resulted in a data subset of 128 channels from 19 subjects performing 11 gestures.

Five single-digit gestures including thumb (D1), index finger (D2), middle finger (D3), ring finger (D4), and little finger (D5) extension, were used for modelling muscle volumes and single label classification. Two double-digit gestures (extension of D1-D2, extension of D2-D3), three triple-digit gestures (extension of D1-D2-D3, extension of D2-D3-D4, extension of D3-D4-D5), and one four-digit gesture (extension of D2-D3-D4-D5) were used for multilabel classification assessment; multi-digit gestures were not included in any modelling step and only used for multi-label assessment, to test for generalizability to completely new gestures.

### Average volume modelling

**Fig. 1:**
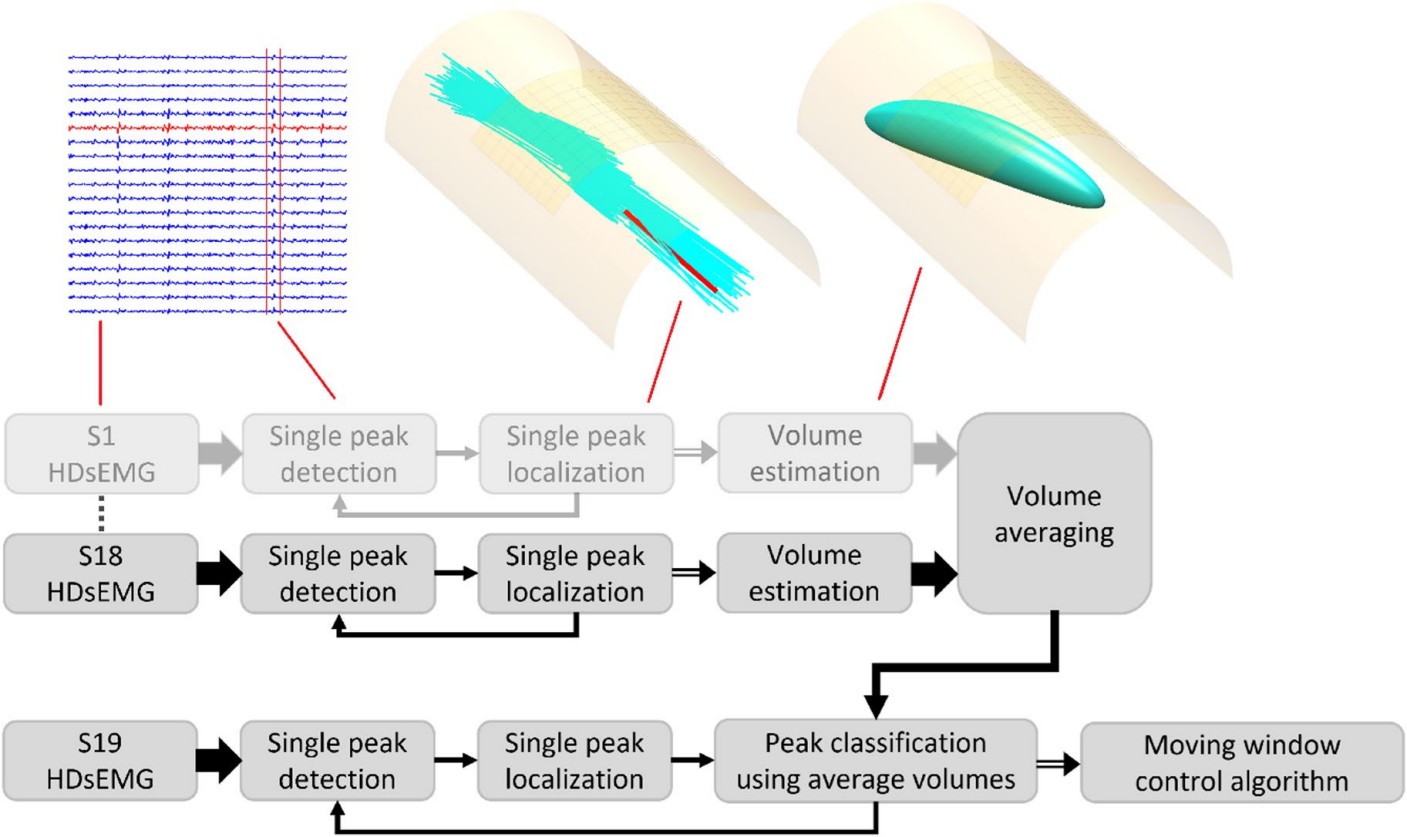
The block diagram illustrates the volume modelling (top row) alongside illustrative figures for different steps noted by red lines, and the gesture classification (bottom row). The faded row indicates a repetition of the same process, and the different arrows conceptually indicate different amounts of information. For volume modelling, time-domain EMG peaks were first detected and localized individually; the distributions of the localized peaks informed the shapes of representative ellipsoid volumes, which were then averaged across 18 of the 19 subjects. For gesture classification, localized EMG peaks, from the subject excluded during modelling, were classified using their position in relation to the average volumes. Gestures were then classified with a moving window which accounts for multiple subsequent peaks.

We used a previously developed method^30^ to generate representative ellipsoid volumes of forearm extensor muscles by localizing numerous time-domain peaks in HDsEMG data. The three-dimensional distributions of localized peaks, for each action, shaped the ellipsoid for each volume representation. These volumes were then used to classify peaks from a different subject, as well as completely new gestures, summarized in Fig. 1.

The localization step was first described for estimation of motor unit positions here^32^, and later applied to individual EMG peaks for volume estimation here^30^. In this method, individual EMG peaks were first identified with peak-detection on a single channel. Centred around each identified peak, a time window of 31 samples (the amplitude values of the signal 15 samples before and 15 samples after the identified peak) was then extracted across all channels. The spatial surface distribution of the EMG peak was plotted using the row and column position for each electrode (as x and y coordinates) and the energy (calculated as the sum of all squares) within the time window at each channel (as z coordinates). A surface Gaussian function was then fitted to these values, which had parameters for maximum peak amplitude and position (Gaussian function centre), as well as distribution width, length, and rotation. The width and rotation of the Gaussian fit informed an estimate of the depth and fibre direction of the MUAP source. In the original method^30^, the Gaussian fit was applied to the peak-to-peak amplitude at each channel, instead of calculating the energy as was done in this work. Compared to using the peak-to-peak values, the signal energy is theoretically less dependent on individual samples, since it is calculated on the full extracted window, and was therefore chosen to improve the stability of position estimates.

**Fig. 2:**
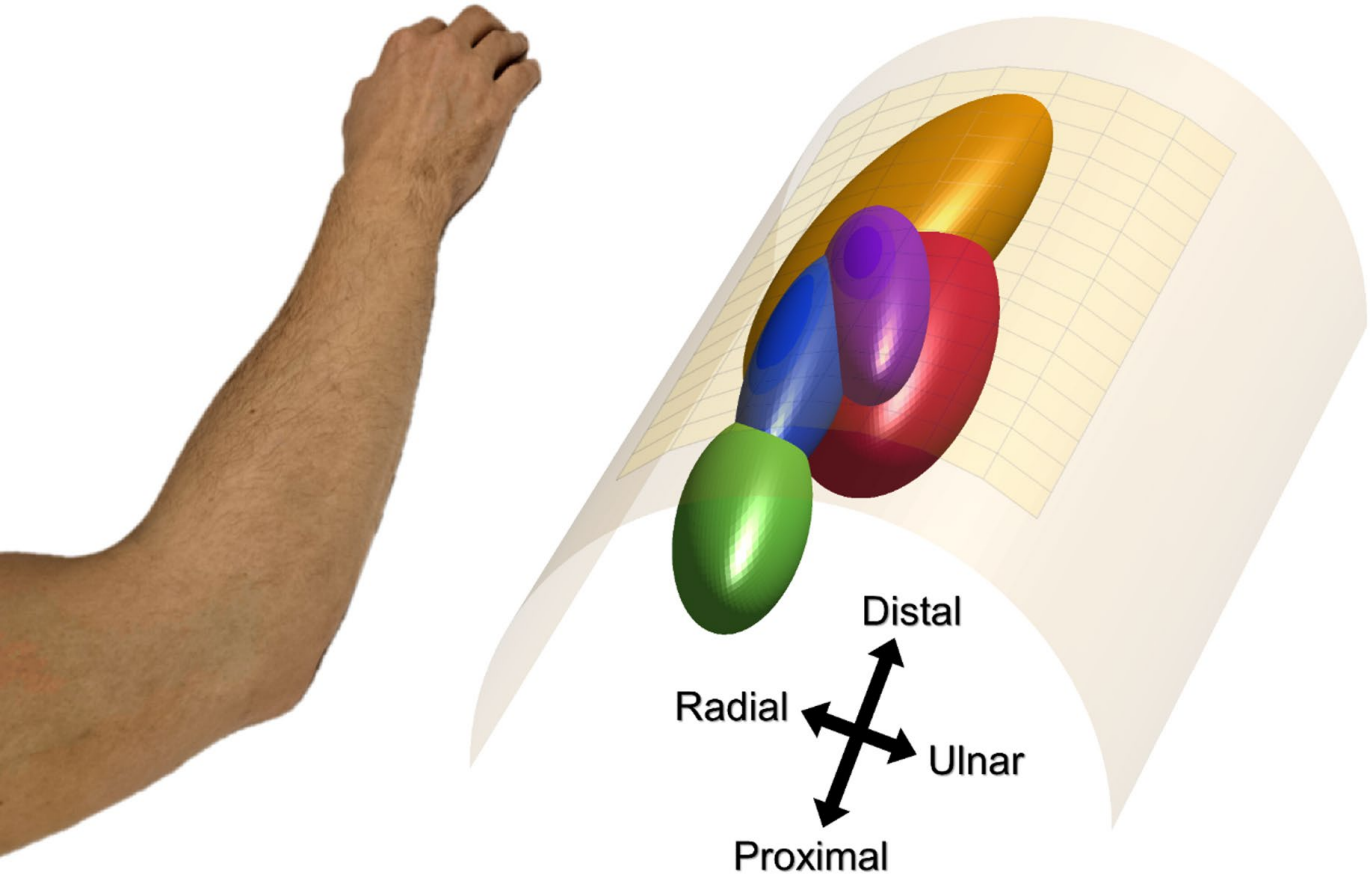
The cylinder model (right) with a set of volume representations, averaged across 18 of the 19 subjects, for extension of the thumb (red), index finger (orange), middle finger (green), ring finger (blue), and little finger (purple). Each volume is defined by an ellipsoid function with the shape determined by the mean spread of localized EMG peaks from the corresponding action. The forearm (left) illustrates the orientation of the model.

In the volume estimation step, five muscle volume representations were estimated, corresponding to the activity during extension of each individual digit, using data from the single digit gestures. For each of the five gestures, the spatial distribution of localized EMG peaks were used to generate the shape of each representative ellipsoid volume; a detailed description of the volume generation can be found here^30^. The mean estimated position and fibre direction was first calculated. Then, the standard deviations were calculated along the fibre direction, perpendicular to the fibre direction, and for the depth. The average volumes used for gesture classification were generated from the average mean and standard deviations across participants for each volume. Since generalizability to completely new individuals was a focus of this work, the averaging was done in a leave-one-out manner, meaning that for each participant a new set of average volumes to be used for classification were calculated from the remaining 18 participants (Fig. 2).

### Classification procedure

#### Ellipsoid based spatial classification

Once the volume models were established, they were used as a reference for classifying new EMG peaks. Ellipsoid based spatial classification was performed in the same manner as in ^30^. Each volume is represented by an iso-surface, a mathematical function which defines a boundary in three-dimensional space using x, y, and z coordinates (Fig. 2). Inserting a specific coordinate into the iso-surface function returns a value which increases with increased distance to the volume centre, modified by the radius of the volume in each direction.

$$\:f\left(x,y,z\right)=\frac{{x}^{2}}{{r}_{x}^{2}}+\frac{{y}^{2}}{{r}_{y}^{2}}+\frac{{z}^{2}}{{r}_{z}^{2}}$$

The estimated position of each EMG peak was inserted into the function representing each volume, returning a set of iso-function values. The EMG peak was classified as belonging to the volume generating the lowest iso-function value. However, different placements of the electrode grids could lead to large offsets between the volumes and the EMG peaks being assessed. Therefore, the volumes were first centred by estimating an offset position which was subtracted for all volumes. The centring offset was calculated from one second of EMG data during ring finger extension. This action was chosen because ring finger extension was found to generate activity in the centre of all volumes with great consistency; the same tendency was also found in ^30^.

#### Moving windowing classification

**Fig. 3:**
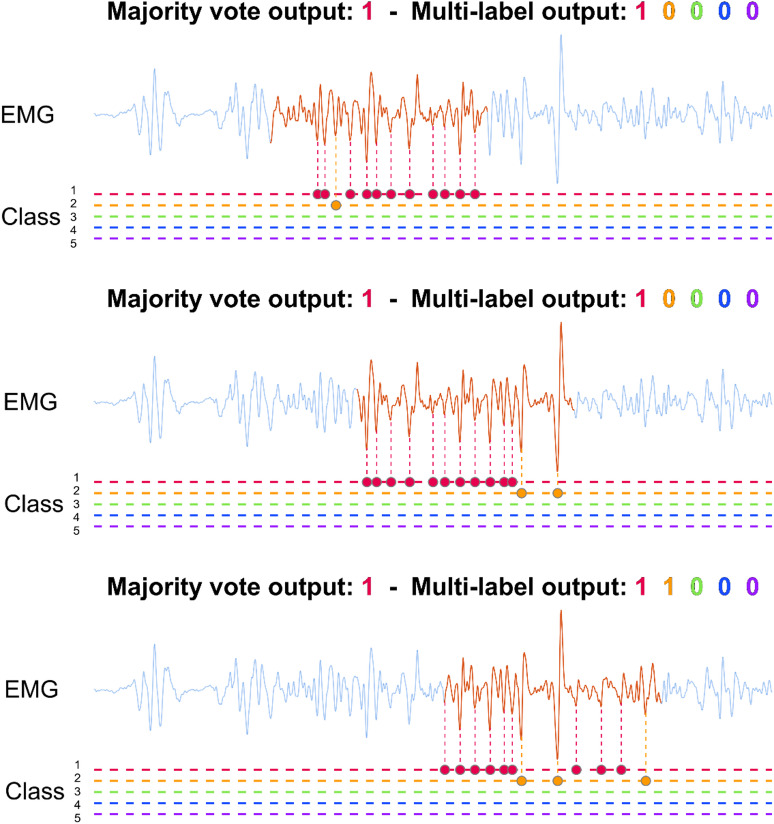
The moving window classification procedure is illustrated on the same EMG channel (light blue lines) overlapped with the 250 ms moving window (red line), for three different segments with a 100 ms stride. The horizontal dashed lines represent the five classes. The coloured dots denote the identified peaks on the line of their assigned class. Using single label majority voting, all cases in this example were classified as thumb extension (red). In the multi-label case, with a minimum threshold of 3 peaks, the last window was classified as both thumb (red) and index (orange) extension.

Gesture classification performance was assessed by imitating real-time conditions with a moving window (250 ms window size, 100 ms stride, Fig. 3) by segmenting the EMG data. In the single-label classification test, EMG peaks within each window were classified individually, and the class with the most peaks was labelled as active. For the multi-label classification test, EMG peaks within each window were again classified individually, and any class with three or more assigned peaks was labelled active. This threshold number of peaks was selected post-hoc via a grid search of the threshold number and the moving window size, optimizing for the lowest Hamming loss.

### Evaluation methods

#### True and false positive rates

For both single label and multi-label gesture classification, performance was evaluated using a table of true positive rates (TPR) and false positive rates (FPR), which refers to the percentage of correctly labelled segments and the percentage of incorrectly labelled segments, for each gesture. In the single label task, each of the five single-digit gestures had one corresponding volume and four non-corresponding volumes, which generated one TPR value and four FPR values per gesture. In the multi-label task, all 11 gestures were included and the number of corresponding volumes changed depending on the gesture. This allowed for a more thorough examination of individual gestures, the distribution of classification errors, and potential limits in the multi-label classification task.

From anatomical knowledge of the forearm, the extensor pollicis longus (EPL) is located beneath the extensor digitorum communis (EDC), extensor digiti minimi (EDM), and extensor indicis proprius (EIP) muscles. Thus, it is expected that the volume representing the EPL would interfere the most with gesture classification performance. To assess this impact on performance, modelling and single label classification was additionally done after excluding thumb extension, generating one TPR value and three FPR values per gesture.

#### Generic performance metrics

For a wholistic assessment of gesture classification performance, generic metrics were calculated for the multi-label case. The number of true positives, false positives, true negatives, and false negatives, denoted by $\:tp$, $\:fp$, $\:tn$, and $\:fn$ respectively, were calculated for each label separately and added together, as in ^4^.

Hamming loss is defined as the ratio of false estimated labels to the total number of labels in the dataset, $\:Hamming\:loss=\frac{fp+fn}{tp+tn+fp+fn}$. Thus, a lower Hamming loss corresponds to better performance.

Precision is defined as the ratio of true positives to the total number of positive estimated labels, $\:Precision=\frac{tp}{tp+fp}$. This metric is favoured by ‘cautious’ or ‘strict’ classification algorithms, which have a high threshold for generating a positive label, resulting in a minimal number of false positives.

Recall (i.e., true positive rate or sensitivity) is defined as the ratio of true positive estimated labels to the total number of ground truth positive labels, $\:Recall=\frac{tp}{tp+fn}$. Recall is favoured by ‘heedless’ or ‘lenient’ classification algorithms, which have a low threshold for generating a positive label, thus identifying most positive labels while disregarding the number of false positives.

Precision and recall tend to be inversely related, in the sense that optimizing for the performance of one is often to the detriment of the other. The F1-score provides a compromise between precision and recall, $\:{F}_{1}score=2\cdot\:\frac{Precision\cdot\:Recall}{Precision+Recall}$.

## Results

### Single label gesture classification

#### True and false positive rates

Single label TPR and FPR for gesture classification with five classes are summarized in Table 1. The values show the cross-subject means and standard deviations after calculating individual performances in a leave-one-out manner, where the assessed individual is not included in the generation of the model. Note that for the single label classification task, the sum of each row is 100%. The TPRs (diagonal grey elements) were highest for ring finger extension with a cross-subject mean of 95.1%, which was expected since one second of this gesture was also used for centring the volume models. The TPRs were the lowest for little finger extension with a cross-subject mean of 61.9%, largely due to misclassification as thumb extension as seen in the bottom row in Table 1, where off-diagonal elements show FPRs for each volume. Similarly, a large degree of misclassification occurs between thumb and index finger extensions, which indicates that the thumb extension interferes the most with classification performance. Table 2 shows the true and false positive rates after removing the thumb extension gesture from the set.

**Table 1:** Cross-subject mean and standard deviation for true positive rates (diagonal elements) and false positive rates (off-diagonal elements) for 250 Ms windowed moving mode.

	True and false positive rates (%)				
Action	Vol 1	Vol 2	Vol 3	Vol 4	Vol 5
Thumb	76.9 ± 29.9	21 ± 30.2	0.3 ± 1.1	0 ± 0	1.4 ± 4.2
Index	24.1 ± 34	64.1 ± 39.8	6.1 ± 22.2	5.6 ± 18.1	0.2 ± 0.8
Middle	2 ± 8.6	0 ± 0	80.5 ± 35.1	12.2 ± 28.9	5.3 ± 22.9
Ring	0.9 ± 3.1	1.7 ± 6.6	2.2 ± 4	95.1 ± 12.6	0.2 ± 0.6
Little	20.8 ± 29	12.8 ± 31	1.4 ± 4.4	3 ± 10.7	61.9 ± 39.3

**Table 2:** True positive rates (diagonal elements) and false positive rates (off-diagonal elements) for 250 Ms windowed moving mode after excluding thumb extension.

	True and false positive rates (%)			
Action	Vol 2	Vol 3	Vol 4	Vol 5
Index	86.2 ± 30.2	6.2 ± 22.1	6.1 ± 20.2	1.4 ± 5.1
Middle	0 ± 0	82.4 ± 35.4	12.4 ± 29.4	5.3 ± 22.9
Ring	2.2 ± 8.8	2.5 ± 4.6	95 ± 12.4	0.3 ± 1
Little	17.6 ± 36.8	1.2 ± 4.2	3.3 ± 10.2	77.9 ± 37.4

### Multi-label gesture classification

#### True and false positive rates

Multi-label true and false positive rates for gesture classification with 11 classes are summarized in Table 3. The values show the cross-subject means and standard deviations in a leave-one-out manner, similar to the single label test. In contrast to the single-label test, multiple labels could be positive for each segment, and the sum of each row is no longer 100%. This is exemplified by comparing little finger extension in Tables 1 and 3, where a substantial increase in both TPR and FPR is seen for the multi-label task.

Note here that assessments of the bottom six multi-digit gestures were performed on participants not included in the modelling step and, more importantly, were completely new gestures; modelling only made use of data from single-digit extensions. Although the TPRs (grey elements) vary greatly, the highest TPR is much greater than the FPR. For each gesture, even the lowest TPR is greater than the highest FPR in most cases, revealing clearly distinct distributions between actions.

**Table 3:** True positive rates (bold elements) and false positive rates (normal elements) for 11 gestures in the multi-label gesture classification task, with a moving window of 250 Ms and a minimum threshold of 3.

	True and false positive rates (%)				
Action	Vol 1	Vol 2	Vol 3	Vol 4	Vol 5
D1	**75.5 ± 27.1**	29.6 ± 36.1	1.2 ± 3	0 ± 0	6.6 ± 19.5
D2	29.8 ± 34.7	**74.8 ± 34.2**	10.5 ± 26.8	12 ± 28.8	2.1 ± 7.8
D3	6.7 ± 21.1	1.1 ± 3.8	**90.7 ± 25.5**	22.2 ± 39.4	5.3 ± 22.9
D4	6.1 ± 13.9	7.4 ± 18.1	16.3 ± 16.1	**97.3 ± 7.6**	2.4 ± 6.1
D5	38.5 ± 38.1	21.7 ± 35.5	3 ± 8.5	8.1 ± 19.1	**77.5 ± 34.5**
D1, D2	**69.6 ± 30.9**	**52.8 ± 40**	6.7 ± 22.2	1.4 ± 3.8	3.7 ± 13.9
D2, D3	8.4 ± 20.6	**19.8 ± 25.9**	**82.2 ± 29**	34.5 ± 37.4	3.7 ± 16.3
D1, D2, D3	**33.3 ± 31.4**	**25.4 ± 28.8**	**74.8 ± 36**	6.6 ± 17.6	2.4 ± 5.2
D2, D3, D4	12.6 ± 25.9	**13.9 ± 28.4**	**73.3 ± 32.3**	**76.6 ± 29.4**	10.4 ± 27.1
D3, D4, D5	18 ± 24.4	19.2 ± 34	**51.6 ± 32.2**	**79.9 ± 28.3**	**19.7 ± 30.2**
D2, D3, D4, D5	19 ± 20.5	**26 ± 33**	**66.7 ± 32.7**	**77.9 ± 24.8**	**11 ± 26.2**

#### Multi-label performance metrics

**Fig. 4:**
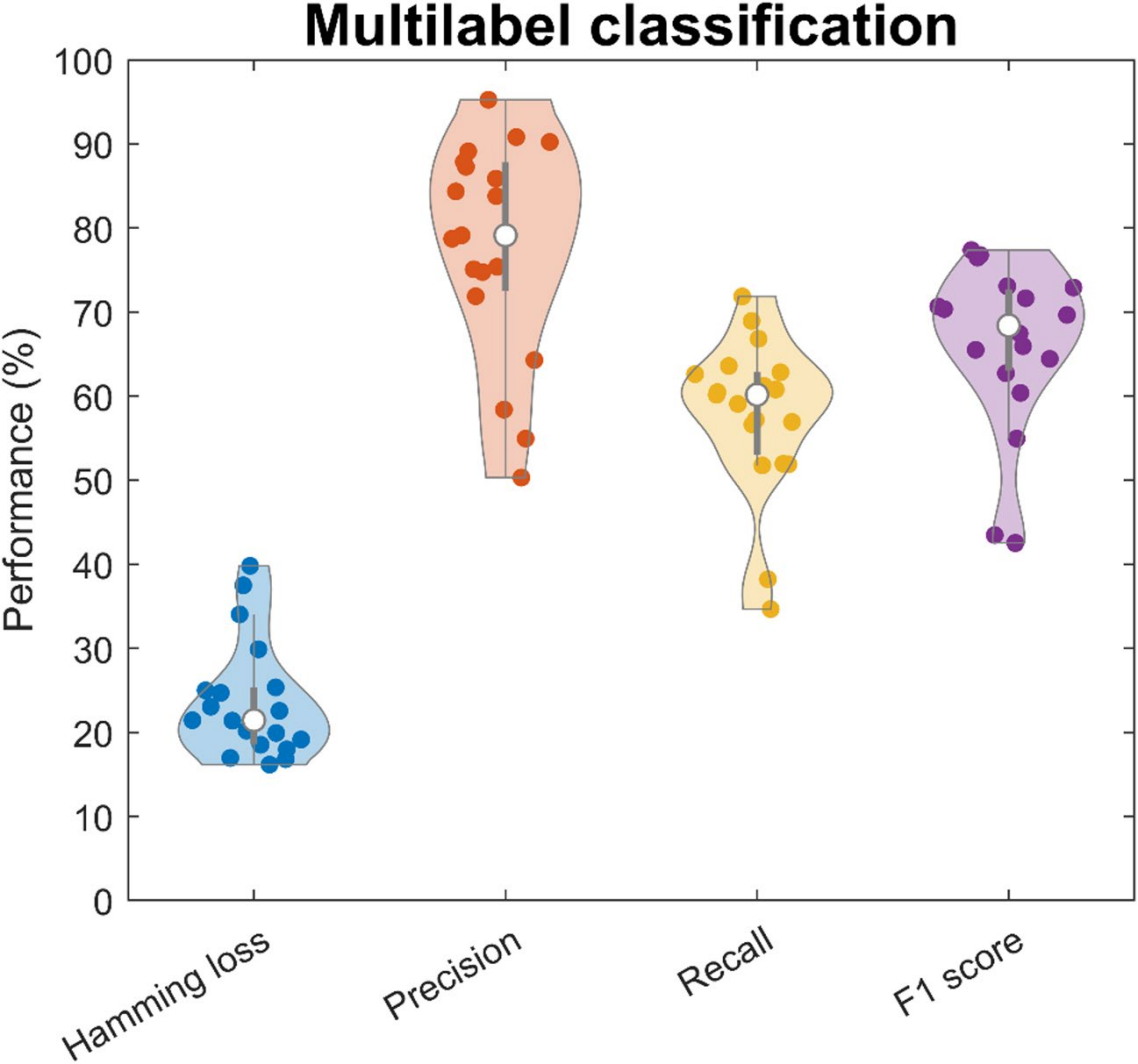
A violin plot showing performance metrics for multi-label gesture classification. For Hamming loss, lower is better; for precision, recall, and F1-score, higher is better. Coloured dots represent individual subject performances; white dots indicate the mean. Shaded regions illustrate the approximate data distribution, with visible skew due to a few low-performing outliers.

The generic performance metrics are summarized in a violin plot (Fig. 4). The results show a cross-subject mean Hamming loss of 23.7, Precision of 77.8, Recall of 57.8, and F1-score of 66.0%; with a standard deviation Hamming loss of 7.0, Precision of 12.9, Recall of 9.3, and F1-score of 9.9%; a median Hamming loss of 21.4, Precision of 79.1, Recall of 60.1, and F1-score of 68.4%; with interquartile range Hamming loss of 18.7–25.3, Precision of 72.6–87.7, Recall of 53.1–62.8, and F1-score of 63.1–72.6%. The better median performance compared to mean performance suggests that the distribution of performances is skewed by low performance outliers. Precision performance is comparatively much higher than recall performance, which indicates that the method is ‘strict’. The lower recall performance indicates difficulties in correctly identifying all positive labels at the same time, and that a larger window or lower threshold can result in a more balanced performance.

## Discussion

This study presents a novel approach to finger gesture classification based on representative muscle volume modelling, emphasizing generalizability. By functional modelling of the active regions in the forearm during extension of individual digits, averaged across multiple subjects, representative volumes were generated for the corresponding muscles. Using these representative volumes, we demonstrated the feasibility of a real-time classification algorithm that generalizes across users and even to new gestures. By leveraging a leave-one-out approach to modelling and testing, the study showed that functional volume representations of muscles generalize across most individuals, which makes this modelling approach suitable for widespread applications. The presented method is an initial step towards the creation of comprehensive three-dimensional models, usable by anyone for gesture classification without additional training or modelling data. In contrast, neural network approaches^4,6,19,33^ require vast amounts of training data, are comparatively computationally demanding, and typically lack explainability. The representative volume modelling approach in this study, provides a visually intuitive, explainable, and conceptually simple alternative to prior methods.

The study also demonstrated a limited degree of generalizability to completely new gestures, as seen in Table 3. Notably, EMG data from multi-digit gestures was never included in the functional modelling of muscle volumes. The multi-digit gestures were instead classified using volumes generated by single-digit gestures from other participants only. The aim was to test the limits of generalizability in a representative anatomical model, and to the best of our knowledge, other methods have not been tested in this manner. Table 3 demonstrates that the EMG data from each multi-digit gesture was predominantly classified as belonging to the volumes corresponding to the active digits (grey elements). The limitation of this generalizability was seen in the overall recall rate, summarized in the violin plot (Fig. 4). The limited recall rate suggests that there were difficulties in identifying multiple muscles at once. The moving window classification used in the multi-label test required three or more EMG peaks to be classified to a volume to label it as positive. This may severely limit performance for gestures with many simultaneously active muscles, since each EMG peak was assigned to only one volume. In these cases, a longer time window or lower thresholds might be required when identifying, e.g., four simultaneously active digits. Another explanation for the limited recall performance might be that the shape or structure of the muscles were significantly different when multiple digits contracted simultaneously compared to individual digit contractions. Thus, the question remains whether sufficiently complex gesture compositions are possible to identify at all.

The method for generating volume representations of muscles^30^ was previously only demonstrated on single-digit gestures with inter-session modelling on a dataset tailored to generate optimal volumes using stable isolated isometric contractions. This study further validated the method by successfully applying it to a generic publicly available HDsEMG dataset^31^. This indicates that the method is easily applied to other HDsEMG datasets; however, it should be applied to a wider range of datasets to gauge any potential limitations. Comparable studies in the literature using the “Hyser” dataset were to our knowledge limited, since the test case is very specific, focusing on the classification of finger extensions only. However, similar leave-one-out approaches in cross-subject classification have been used for various generic gestures and wrist movements. E.g., Shi et al. tested convolutional neural networks with and without transfer learning techniques, using 10 generic gestures, achieving true positives rates between 65 and 88% without transfer learning and 80 and 95% with transfer learning^34^. Whereas the single-label true positive rates in this study were between 61.9 and 95.1% (Table 1). However, it is difficult to draw strong conclusions due to differences in protocol, including data segmentation and gesture subsets. In theory, classifying individual finger extensions should be more difficult than wrist movements, since the EDC occupies a comparably limited region, in contrast to the multiple wrist muscles throughout the forearm. On the other hand, classification is also more difficult using 10 classes compared to 5. Although the purpose of this study was to test the feasibility volume representations in gesture classification, in the future, the method needs to be tested alongside other techniques under identical conditions to further gauge its performance.

Another finding of this study was the amount of overlap in the single-digit test between thumb extension and both index and little finger extension, while thumb extension showed almost no overlap with middle and ring finger extension. This provides further insights into the functional anatomy of the dorsal compartment of the forearm; however, further studies are required to fully explain this discrepancy. It is worth noting that while the EDC is a fused muscle extending all fingers, previous studies (as well as this study) demonstrated a specific segmentation of the EDC where middle finger (EDC-3) extension was mainly generated by EMG activity at the proximal end of the EDC muscle^30,35–37^. In contrast, the EDC segments responsible for index finger (EDC-2) extension and little finger (EDC-5) extension seem to be located at the distal end. However, it is important to note that extension of the index and little finger are both controlled by two muscles, EDC-2 and EIP; EDC-5 and EDM respectively. It is unclear to what extent this affects the generated volume representations and thus the gesture classification performance; a more comprehensive model could include multiple volumes for these actions. Thus, the results indicate that the EPL is mainly located beneath EDC-2 or EIP, and EDC-5 or EDM, which is also seen in Fig. 2. While the volume representing the EPL was deeper than the other volumes, as expected anatomically, the current method may have potentially underestimated the differences in depth, leading to an increased overlap between volumes. In the localization step, increasing the separability of estimated positions along the depth axis is a potential avenue for improvement. Multilayered volume conductor models^38,39^, or conductor models based on anatomical scans^40^, i.e. magnetic resonance imaging, might improve upon this aspect, by more accurately reflecting the underlying structure and its conductive properties.

Another limitation of the current method was the lack of a ‘rest’ or ‘other’ class. The current method assigned each EMG peak to one of the volumes in the model. Thus, co-contraction from, e.g., wrist muscles could have been falsely classified as one of the digit extensors. A possible solution could be to identify some maximum distance threshold for ellipsoid classification. EMG peaks localized too far away from any of the volumes in the model would then be excluded. As previously mentioned, wrist extensors were not included to avoid overcomplicating the model. However, with sufficiently isolated contractions during model generation, we predict that these muscles are more easily separated from the finger extensors since they are anatomically distinct (whereas the EDC is a fused muscle). Adding more muscles to the same model could, however, lead to performance limitations as more muscles compete in the classification process. Thus, we distinguish between two different potential limitations: the separability of muscle volumes, and the competition in classification that arises when increasing the number of included muscles. By excluding the wrist extensors, we simplified the analysis in order to focus on the separability of muscle volumes. Whether the inclusion of a large number of muscles poses a significant problem, and how to circumvent it, needs to be explored in the future.

The focus in this paper was on generic HMIs with healthy participants. Another important area of application is the control of prosthetic limbs. In these cases, individual variability is very high, which likely limits or prevents cross-subject generalizability. However, we believe that intra-subject generalizability to new gesture permutations would remain unaffected. Generating a unique model for each amputee, of their unique anatomical organization might be the best solution. As for other injuries or diseases, such as stroke or spinal cord injury, cross-subject generalizability may be less affected; if the underlying anatomy remains the same, with only impaired muscle strength, then this method could retain the ability to identify which muscles are active in on-off classification tasks. For regression tasks or multiple force levels, however, further subject specific calibration would still be required.

Another form of generalizability is that of different arm positions, which was not studied. Accounting for, e.g., the rotation of the forearm remains a difficult challenge to solve as the underlying muscle organization can shift drastically. For a study on this topic, a comprehensive dataset is needed with repeated recordings at multiple positions. There are two potential solutions for this problem that comes to mind, which should be explored. Firstly, a simple positional shift of the model, as a function of arm rotation. This approach could be suitable if the forearm retains enough of its relative organization between muscles as it rotates. A more complicated solution would be to estimate a transform function, which can be applied to the coordinate space for various rotation angles. This approach has the potential to account for stretching and twisting of the underlying structure, while remaining sufficiently simple to implement into the current method. Additionally, external forces, such as gravity, also affect the underlying muscle activity. While this may not affect the underlying muscle organization, it could create a form of bias in the contraction patterns for different orientations. If these effects are small, it could potentially be enough to select proper thresholds for identifying activations. A more comprehensive solution would require tracking these external forces in relation to the arm and compensating the control algorithm accordingly, which might ultimately be specific to each unique application.

Furthermore, while the focus of this paper was real-time gesture classification, other promising applications exist as well. Most notably, the method can be used to estimate the distribution of EMG activity between muscles, which could be of great value in rehabilitation assessments. For example, during rehabilitation exercises, the distribution of EMG activity could provide a measure for muscle compensation from co-contracting muscles. Feedback on the degree to which intended and unintended muscles are being used could inform therapists and patients of the quality of current exercises. Such quantitative assessment tools are in high demand, since rehabilitation therapists often rely on their own past professional experience to make assessments^41^. The presented method could thus provide valuable information, presented in an intuitive manner, requiring minimal training or expertise.

## Data Availability

The datasets used and/or analysed during the current study are publicly available (https://doi.org/10.13026/ym7v-bh53, pr_dataset, session1, maintenance_raw, using the toolbox script “main_pr.m”). The MATLAB code used for the method is available on GitHub. https://github.com/Neuroengineering-LTH/MATLAB-Decomposition-localization-and-modelling-of-HDsEMG.
